# Changes in Neurocognitive Architecture in Patients with Obstructive Sleep Apnea Treated with Continuous Positive Airway Pressure

**DOI:** 10.1016/j.ebiom.2016.03.020

**Published:** 2016-03-25

**Authors:** Ivana Rosenzweig, Martin Glasser, William R. Crum, Matthew J. Kempton, Milan Milosevic, Alison McMillan, Guy D. Leschziner, Veena Kumari, Peter Goadsby, Anita K. Simonds, Steve C.R. Williams, Mary J. Morrell

**Affiliations:** a Sleep and Brain Plasticity Centre, Department of Neuroimaging, IoPPN, King's College and Imperial College London, UK; b Danish Epilepsy Centre, Dianalund, Denmark; c Sleep Disorders Centre, Guy's and St Thomas' NHS Foundation Trust, UK; d Academic Unit of Sleep and Breathing, National Heart and Lung Institute, Imperial College London, UK; e NIHR Respiratory Disease Biomedical Research Unit at the Royal Brompton and Harefield NHS Foundation Trust, Sydney Street, London SW3 6NP, UK; f Department of Psychology, IoPPN, King's College London, UK; g Department of Basic and Clinical Neuroscience, IoPPN, King's College London, UK; h IoPPN, King's College London, UK; i Department for Environmental and Occupational Health, University of Zagreb, School of Medicine, Andrija Štampar School of Public Health, Zagreb, Croatia

**Keywords:** Obstructive sleep apnea, Continuous Positive Airway Pressure, Neuroimaging, Cognition

## Abstract

**Background:**

Obstructive sleep apnea (OSA) is a chronic, multisystem disorder that has a bidirectional relationship with several major neurological disorders, including Alzheimer's dementia. Treatment with Continuous Positive Airway Pressure (CPAP) offers some protection from the effects of OSA, although it is still unclear which populations should be targeted, for how long, and what the effects of treatment are on different organ systems. We investigated whether cognitive improvements can be achieved as early as one month into CPAP treatment in patients with OSA.

**Methods:**

55 patients (mean (SD) age: 47.6 (11.1) years) with newly diagnosed moderate–severe OSA (Oxygen Desaturation Index: 36.6 (25.2) events/hour; Epworth sleepiness score (ESS): 12.8 (4.9)) and 35 matched healthy volunteers were studied. All participants underwent neurocognitive testing, neuroimaging and polysomnography. Patients were randomized into parallel groups: CPAP with best supportive care (BSC), or BSC alone for one month, after which they were re-tested.

**Findings:**

One month of CPAP with BSC resulted in a hypertrophic trend in the right thalamus [mean difference (%): 4.04, 95% CI: 1.47 to 6.61], which was absent in the BSC group [− 2.29, 95% CI: − 4.34 to − 0.24]. Significant improvement was also recorded in ESS, in the CPAP plus BSC group, following treatment [mean difference (%): − 27.97, 95% CI: − 36.75 to − 19.19 vs 2.46, 95% CI: − 5.23 to 10.15; P = 0.012], correlated to neuroplastic changes in brainstem (r = − 0.37; P = 0.05), and improvements in delayed logical memory scores [57.20, 95% CI: 42.94 to 71.46 vs 23.41, 95% CI: 17.17 to 29.65; P = 0.037].

**Interpretation:**

One month of CPAP treatment can lead to adaptive alterations in the neurocognitive architecture that underlies the reduced sleepiness, and improved verbal episodic memory in patients with OSA. We propose that partial neural recovery occurs during short periods of treatment with CPAP.

## Highlights

• One month of CPAP treatment leads to adaptive alterations in the neuroanatomy and neurocognition in patients with OSA.
• Improvements in sleepiness and verbal episodic memory were demonstrable following only one month of treatment with CPAP.
• Our findings support the clinical rationale for the use of CPAP treatment to relieve sleepiness, even for a short duration.

Obstructive sleep apnea (OSA) is a highly prevalent sleep disorder that has been linked with dementia, stroke and increased risks of cardiovascular disease. Continuous positive airway pressure (CPAP) treatment has been shown to be effective in reducing sleepiness in moderate to severe OSA. However, the evidence for its use to reverse or slow the rate of cognitive decline is unclear. Our findings show that one month of CPAP treatment in patients with moderate to severe OSA leads to adaptive alterations in the brain networks associated with reduced sleepiness and improved memory.

## 1 Introduction

Obstructive sleep apnea (OSA) is a debilitating, chronic multisystem sleep disorder that arises from recurrent partial or complete pharyngeal obstruction during sleep (Lévy et al., 2015, Malhotra et al., 2015). It has been proposed to have an important, if not fully understood, bidirectional relationship with several major neurological disorders (Lévy et al., 2015, Rosenzweig et al., 2015, Rosenzweig et al., 2014). A close association of OSA with early onset of cognitive decline, by as much as a decade, has been reported, whilst a growing body of clinical and animal work advocates that OSA should be recognized as one of the rare modifiable risks for Alzheimer's dementia (Rosenzweig et al., 2015, Osorio et al., 2015, Yaffe et al., 2014). In addition, treatment with Continuous Positive Airway Pressure (CPAP), the main treatment for OSA, has been also variably shown to halt the onset, decelerate the progression, or offer a better prognosis in patients with co-morbid dementia, epilepsy and stroke (Osorio et al., 2015, Yaffe et al., 2014, Campos-Rodriguez et al., 2014, Pornsriniyom et al., 2014, McMillan et al., 2015).

Numerous clinical studies over the years have demonstrated changes in the central nervous system (CNS) of patients with OSA, including altered resting cerebral blood flow pattern (Baril et al., 2015) with hypoperfusion during the awake states (Joo et al., 2007), changes in the electroencephalogram (EEG) and aberrant cortical excitability (Morisson et al., 1998, Morisson et al., 2001, Dingli et al., 2002) and changes in both white and gray matter (Zimmerman and Aloia, 2006, Torelli et al., 2011, Kumar et al., 2014, Morrell and Glasser, 2011, Macey et al., 2008). These studies have largely also suggested a putative neurocircuitry fingerprint at which core lies the disconnection of the frontal regions (O'Donoghue et al., 2012) and the disruption of the (cerebello)-thalamocortical oscillator with involvement of the hippocampal formation (Rosenzweig et al., 2013a, Rosenzweig et al., 2014, Torelli et al., 2011, Yaouhi et al., 2009).

The additive impact of progressive changes in sleep quality and structure, changes in cerebral blood flow, neurovascular and neurotransmitters, plus the cellular redox status are all likely to contribute to the cognitive deficits reported in up to one out of four newly diagnosed OSA patients (Rosenzweig et al., 2015, Lavie, 2015, Antonelli Incalzi et al., 2004). Despite concerted efforts, OSA remains widely underdiagnosed in the general population, with its prevalence predicted to increase sharply over the coming years due to the epidemics of aging and obesity (Lévy et al., 2015, Heinzer et al., 2015). The important questions of what, who and when to treat, are far from clear (Rosenzweig et al., 2015, Djonlagic et al., 2015, Dalmases et al., 2015). Persistent deficits, even after prolonged treatment with CPAP in some patients, suggest that early detection of the central nervous system (CNS) sequelae in OSA could be crucial (Rosenzweig et al., 2015, Castronovo et al., 2014, Kylstra et al., 2013).

In a recent study of patients with OSA, augmentation of subjective experience, attention and vigilance has been demonstrated after only one night of CPAP (Djonlagic et al., 2015). However, no appreciative impact on procedural memory consolidation was noted, suggesting differential impact on brain structures underlying these processes (Djonlagic et al., 2015). On the other hand, in a seminal study, three months of CPAP treatment led to a significant recovery of cognitive and morphometric deficits (Canessa et al., 2011). Taken together, empirical clinical experience and early research findings suggest that subjective memory improvements are reported as early as one month following the commencement of CPAP treatment (McMillan et al., 2015). In the present study, we set out to investigate this time frame, testing the hypothesis that one month of CPAP treatment would lead to cognitive improvements, and that any changes would be associated with neuroplastic changes in patients with OSA.

## 2 Methods

### 2.1 Participants And Design

Patients with newly diagnosed OSA (18–65 years old) were recruited from Royal Brompton and Harefield Hospitals' sleep clinics. Inclusion criterion was an apnea/hypopnea index (AHI) > 10 events/h (McMillan et al., 2014). Apneas were defined as > 80% drop in airflow for 10 s. Hypopneas were defined as > 50% reduction in airflow from baseline with a > 4% dip in saturation, or an arousal from sleep (Berry et al., 2012a, Berry et al., 2012b, Rosenzweig et al., 2013b). Exclusion criteria were a history of respiratory, cerebrovascular and/or ischemic heart disease, diabetes mellitus, neuropsychiatric or neurological disorder, alcohol, drug abuse, or psychoactive medications.

The same exclusion criteria were used for healthy controls (age- and education-matched) recruited from a database of healthy volunteers. Those with a history of sleep problems on questionnaires, or evidence of OSA on pulse-oximetry (ODI > 5 events/h) were excluded.

**Fig. 1:**
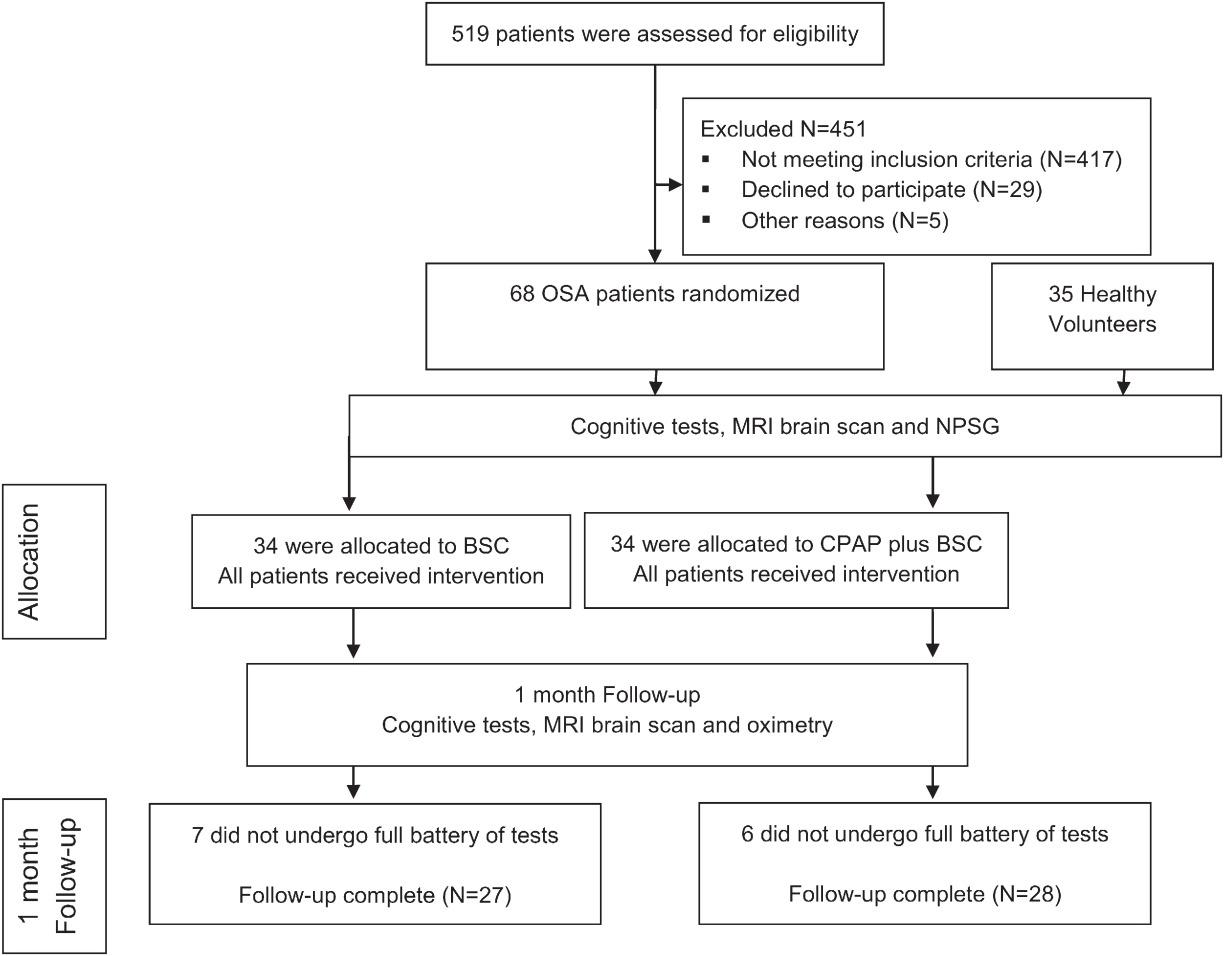
CONSORT diagram.

All enrolled patients were randomly allocated [stratified by age, OSA severity (oxygen desaturation index (ODI) & AHI) and years of education], by an independent study coordinator, to receive CPAP with best supportive care (BSC), or BSC alone, for one month and then re-assessed (Fig. 1).

The study was part of an ongoing research to investigate the impact of OSA on the brain and was approved by the UK central research ethics committee (10/H0706/51). All patients gave written informed consent.

### 2.2 Intervention

CPAP treatment was initiated using standard clinical practice at each center (ResMed S9; with humidification as required).

BSC comprised of advice on minimizing daytime sleepiness through sleep hygiene, naps, caffeine, exercise and weight loss as appropriate to each patient. Both groups were provided with BSC and asked to continue with their usual medical care during the trial.

### 2.3 Assessments

Structured assessments were performed at baseline (patients and controls) and a follow-up after one month (patients only). In addition, all patients received a telephone call at 1 week to record symptoms, side-effects, and to optimize CPAP adherence. All OSA patients completed an inpatient polysomnography (SOMNOscreen PSG, S-Med, UK) prior to CPAP initiation. Domiciliary overnight pulse-oximetry (Konica Minolta Inc.,) was performed at one month. Treatment compliance was measured objectively by download of the CPAP smart card during the one-month visit.

### 2.4 Cognitive Test Battery

Cognitive function of all participants was assessed using a battery of tests comprising the Addenbrooke's Cognitive Examination-Revised (ACE-R) (Mioshi et al., 2006, Graham et al., 2004), Trail Making Test A and B (Reitan, 1979) (TMA, TMB), Logical Memory (LM) Test: immediate and delayed LM with alternate stories used at baseline and follow up, subtests from the Wechsler Memory Scales (Wechsler, 1987), Digit Span Test: forward (DSF) and backward (DSB) (Wechsler, 1987), and Spatial Span subtest forward (SSF) and backward (SSB) (the visual–spatial version of Digit Span) (Wechsler, 1997). The tests used were chosen to target cognitive domains that have been previously shown to be affected by OSA, by our group and other groups (Rosenzweig et al., 2015, Twigg et al., 2010).

ACE-R is a brief battery that provides evaluation of six cognitive domains (orientation, attention, memory, verbal fluency, language and visuospatial ability) (Mioshi et al., 2006). It is useful for detecting dementia and mild cognitive impairment, and it is able to distinguish between patients with progressive degenerative disorders and those with affective disorders (Dudas et al., 2005). The total score is 100, higher scores indicate better cognitive functioning, each domain has individual scores and there are age and education dependent norms for the total score as well as for the individual domains (Mioshi et al., 2006). The subscores for the domain of verbal fluency (ACE-R verbal fluency) were used in this study to asses both executive and language abilities (Shao et al., 2014).

The TMT consists of two parts, A and B; it is one of the most widely used neuropsychological tests that provides information on visual conceptual and visuomotor tracking, motor speed, attention and executive functions (Reitan, 1979). It is a timed test and the score represents the amount of time required to complete the task (Reitan, 1979). Performance decreases with increasing age and lower levels of education although normative data is available (Tombaugh, 2004).

Wechsler Adult Intelligence Scale, third edition (WAIS-III) (Wechsler, 1997) is a commonly used commercially available validated assessment of cognitive function. The LM, DSF, DSB, SSF and SSB tests used in our study were subtests of the WAIS-III and administered and scored as recommended by the WAIS-III instruction manual (Wechsler, 1997). LM tests the patient's ability to remember two short stories presented orally (Wechsler, 1997). There is an immediate test and then a delayed recall test after 30 min; it is a measure of verbal memory. Digit Span task is used to measure working-memory's number storage capacity (Wechsler, 1997). Participants are presented with a series of digits and must repeat them back. The length of the longest list a person can remember is that person's digit span. Whilst the participant is asked to enter the digits in the given order in the forward digit-span task, in the backward digit-span task the participant needs to reverse the order of the numbers (Wechsler, 1997). Spatial Span forward and backwards are a measure of visual–spatial processing and working memory (Wechsler, 1997). It is validated in older patients that as cognitive impairment severity increases there is a reduction in the total score. The Spatial Span backward is a more sensitive measure of early cognitive impairment as opposed to the Spatial Span forward which remains relatively stable regardless of the level of impairment (Wiechmann et al., 2011). Spatial Span forward is an attention task, whereas Spatial Span backward is considered a working memory task as it requires holding information in the memory (Wechsler, 1997).

### 2.5 Magnetic Resonance Imaging

All participants underwent MR imaging and T1-weighted MR-images were acquired using a 1.5 T scanner (Magnetom Vision, Siemens Healthcare, Camberley, Surrey, UK) and a 3D MP-RAGE sequence (TI 300 ms, TE 4 ms, in-plane resolution 1.0 × 1.0 mm) with contiguous 2 mm coronal slices. The T1-weighted images were processed, and volumetry was performed using the automated method FreeSurfer, as previously described by our group (Rosenzweig et al., 2013b).

### 2.6 Statistical Analyses

The Kolmogorov–Smirnov test was used to test the normality of distributions. To analyze differences in a variety of demographic parameters between healthy controls and OSA patients, Student's *t*-test was applied (Table 1). All statistical analyses had a 2-tailed α level of < 0.05 for defining significance and were performed by a biostatistician on the statistical software “STATISTICA 10·0” (http://www.statsoft.com).

**Fig. 2:**
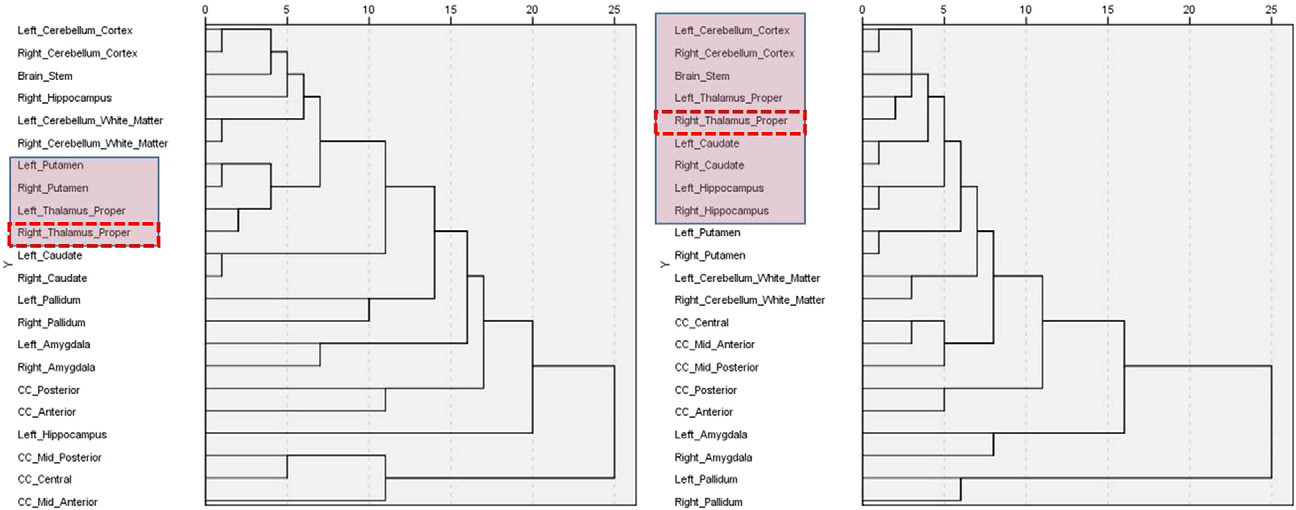
Dendrograms depict relationships between structural changes in brain volumes following one month of intervention with the Continuous Positive Airway Pressure or Best Supportive Care treatment. Dendrograms were constructed using the results of Hierarchical Cluster Analysis (HCA) of the differential ∆ structural changes, using Average Linkage with squared Euclidean distance as a measure of distance (x-axis values). HCA resulted in the formation of clusters that were iteratively joined in a descending order of similarity. Resulting dendrograms show different patterns of neuroanatomical changes in patients with OSA following the interventions with BSC alone (N = 27) from those resulting post CPAP with BSC (N = 28) treatment. The HCA findings also suggest that the thalamic alterations (arrows) in the CPAP group are closely linked to wider core neurocircuitry adaptations (pink boxes).

Investigation of a priori hypotheses was focused on group differences for several neuroanatomical structures that had been previously highlighted by our data and that of other groups, to present network hubs of OSA-affected neurocircuitry: the hippocampus, amygdala, basal ganglia, thalamus, brainstem, corpus callosum (and its subdivisions) and cerebellum (Rosenzweig et al., 2015, Gozal, 2013). In addition, several previously reported affected cognitive domains were assessed (Twigg et al., 2010). The intracranial volume (ICV) calculated by FreeSurfer did not differ significantly between groups (*t*-test, P = .514) and Student's *t*-test was done on the ICV normalized data (i.e. volume/ICV) to assess between-group differences (Supplementary Table S2). Hierarchical cluster analysis (HCA) (Waddell et al., 2014) was further undertaken to identify homogeneous groups of variables among differential (diff ∆) changes in brain volumes following the two interventions. HCA results in the formation of clusters in which profiles are iteratively joined in a descending order of similarity. More specifically, in our study, the agglomerative hierarchical clustering algorithm was used that started with each variable in a separate cluster and combined clusters until only one was left. The resulting cluster hierarchies were displayed as tree diagrams, i.e. dendrograms (Fig. 2). The clustering method was used to show between-groups linkage with squared Euclidean distance as a measure of distance. The final number of clusters has been defined according to the highest coefficient changes in agglomeration schedules. Statistical analysis for HCA was performed with IBM SPSS Statistics version 21.0 (http://www01.ibm.com/software/analytics/spss/).

During post hoc analyses interregional connectivity and inter-domain relationships between the right thalami, somnolence (ESS test scores) and verbal episodic memory (delayed LM test scores) were explored with Pearson correlations; controlled for ICV and normalized for differential changes (diff ∆%) from the baseline (diff ∆ = [V/domain_pre_ − V/domain_post_] / V/domain_pre_ × 100). All results were Bonferroni corrected for multiple comparisons. Finally, partial correlations (Tahmasian et al., 2015) were used to control the body mass index (BMI) confounding effect on correlation coefficients between neuroimaging and cognitive tests' score changes.

## 3 Results

Fifty-five OSA patients (81%) completed the study, in addition to 35 age- and education-matched healthy volunteers (Fig. 1). Attrition was due to incomplete investigations in 10% (7/68) of the BSC group, and 9% (6/68) of the CPAP group. Mean (SD) age of OSA patients was 47.6 (11.1) years, ODI 36.6 (25.2) events/hour, and ESS 12.8 (4.9). Baseline demographic characteristics of participants are shown in Table 1. Baseline sleep characteristics are shown in Supplementary Table S1. The baseline characteristics were similar between the CPAP with BSC group and BSC only group. Median CPAP use over one month was 5.0 (range 1.9–6.9) hours/night (Table 1).

### 3.1 A Priori Hypotheses Investigations

**Fig. 3:**
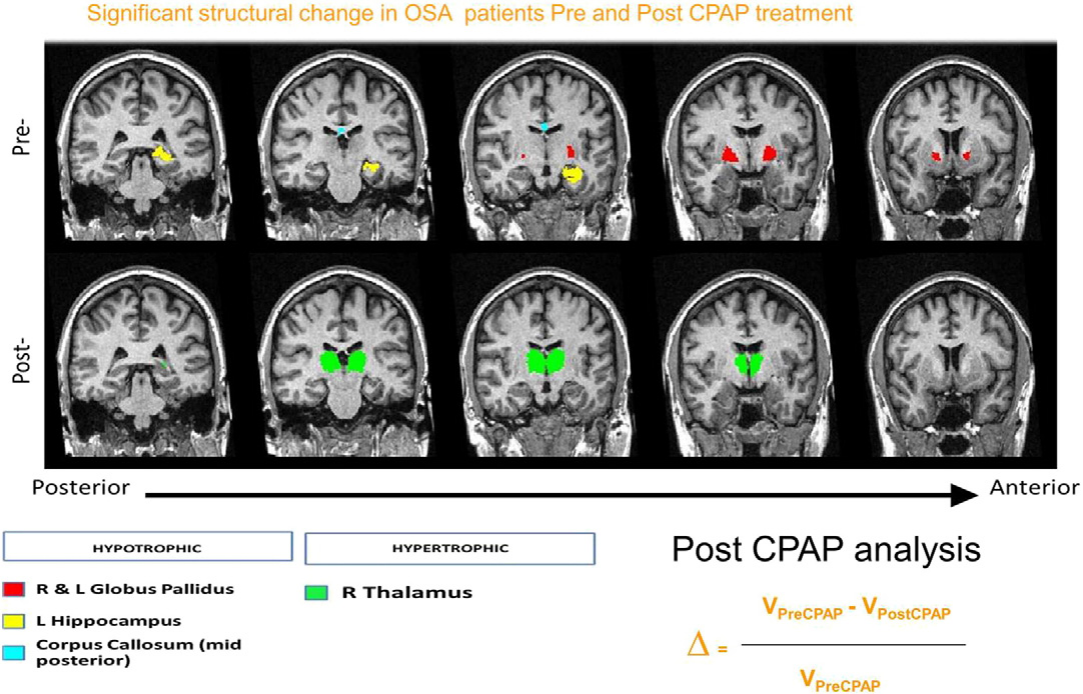
Significant structural change in OSA patients Pre and Post CPAP treatment. The first row (Pre-) shows neuroanatomical structures that were noted as hypotrophic in OSA patients (N = 55) before any intervention, by comparison to matched healthy volunteers (N = 35); bilateral globus pallidi, left hippocampi and mid posterior section of corpus callosum. The second row (Post-) depicts hypertrophic changes noted in bilateral thalami following one month of treatment with CPAP; the longitudinal structural changes were calculated by normalization to the individual baseline volumes (refer to the equation).

At baseline, hypotrophic changes were recorded in the left hippocampus, in the bilateral pallidus and the mid-posterior part of the corpus callosum of OSA patients (n = 55) in comparison to those recorded in healthy controls (n = 35) (Fig. 3: pre-CPAP). Following one month of interventions, the between-group comparison (patient groups vs healthy controls) no longer showed statistically significant hypotrophic changes in either of these structures, suggesting that adaptive neuroplastic changes have occurred (Supplementary Table S2). The hierarchical cluster analysis further demonstrated the distinct alteration pattern for each intervention during this period (Fig. 2), with the thalamic alterations in the CPAP group closely linked to wider core neurocircuitry adaptations, compared to the BSC group.

The differential neuroanatomical structural changes at one month between the CPAP group and the BSC group were subsequently explored. In the CPAP group, a significant hypertrophic trend (Fig. 3) for the right thalamus was recorded [mean diff ∆% (SD): CPAP, 4.04 (13.61) vs BSC, − 2.29 (10.66); P = 0.06]. No other statistically significant differences between groups were found (Supplementary Table S3).

At baseline, OSA patients showed significantly impaired cognitive processing in several domains by comparison to healthy controls (Table 2). Following one month of interventions, improvement was recorded in the majority of tested domains compared to healthy controls (Table 2). However, comparison of differential cognitive changes between interventions, suggested that only changes in sleepiness [mean diff ∆% (SD): CPAP, 2.46 (39.97) vs BSC, − 27.97 (46.46); P = 0.012] and verbal episodic memory test scores [23.41 (32.45) vs 57.20 (75.46); P = 0.037] were statistically significant (Supplementary Table S4).

In summary, the findings of a priori between-intervention analyses demonstrated the differential neuroplastic cognitive and morphometric adaptations already after one month of treatment (Fig. 2, Fig. 3; Supplementary Tables S3, 4).

### 3.2 Post Hoc (Secondary) Analyses

**Fig. 4:**
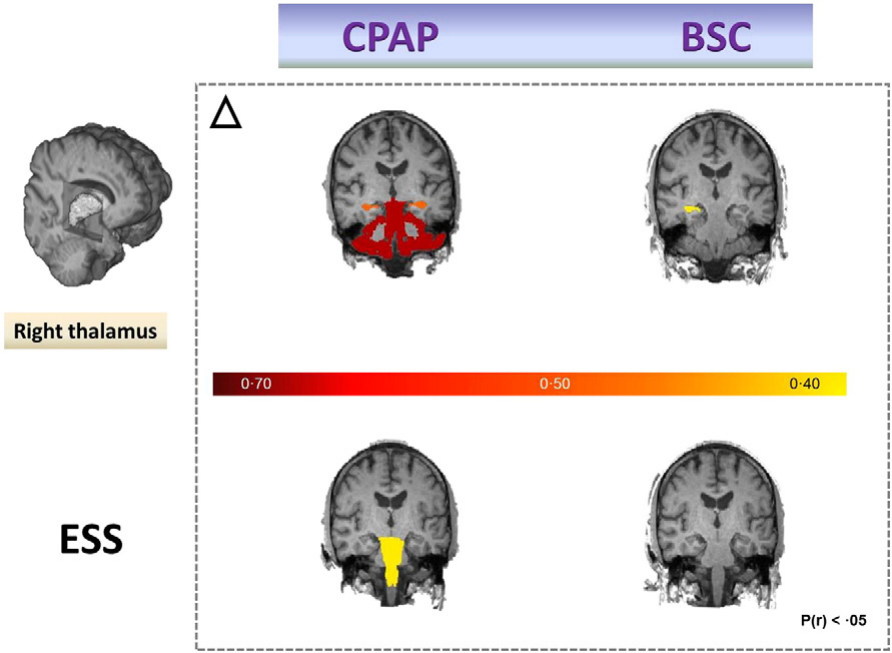
Neuroplastic structural changes following one month of intervention with the Continuous Positive Airway Pressure or Best Supportive Care treatment. Potential strengthening in interregional connectivity between the non-dominant thalamus with hippocampus and (or) cerebellar cortex in OSA patients was noted only in CPAP treated group (first row). In addition, in the same group of patients, improvement in sleepiness (as shown by the ESS), was strongly positively correlated to changes occurring in brainstem during this period (second row).

Secondary correlational analyses were dictated by the three significant findings of a priori investigations. We explored the pathomechanisms behind improvements in episodic memory, somnolence and the hypertrophic trend for the thalamus in the CPAP group (Fig. 3, Fig. 4). Significant correlation between improvement in sleepiness and volumetric changes in the brainstem (r = − 0.37; P = 0.05) was found (Fig. 4), whilst improvements in verbal episodic memory were not obviously correlated to any singular morphometric change. Similarly, hypertrophic changes in the right thalamus appeared strongly correlated to changes in bilateral cortical cerebellar (CC) and hippocampal (H) structures (left CC r = 0.77; P = 1.96 10^− 6^; right CC r = 0.75; P = 3.78 10^− 6^; left H r = 0.49; P < 0.01; right H r = 0.51; P < 0.01), which was not the case for OSA patients that received only BSC intervention. In the CPAP group, thalamic changes were also found to be correlated with the changes in overall cognitive functioning (ACE-R) scores (r = − 0.554; P = 0.002), attentional span changes (DSF, r = − 0.499; P = 0.007) and with the changes in visuospatial working memory (SSB, r = − 0.370; P = 0.05).

Positive correlations between improvements in scores of verbal episodic memory and semantic subdomain of verbal fluency (fluency ACE-R) (r = 0.406; P = 0.032) that were otherwise negatively correlated in the BSC group (r = − 0.190, P = 0.343), were also shown.

Episodic memory showed significant positive correlation with span changes in the CPAP (r = 0.451, P = 0.02) vs BSC group(r = − 0.081, P = 0.69). ESS scores were not significantly correlated to changes in working memory in the CPAP (r = 0.295, P = 0.127), although they were significant in the BSC group (r = 0.591, P = 0.001). The correlations between noted changes over one month and ODI values of patients have been further explored; here, a significant correlation between changes in the anterior part of corpus callosum and ODI (r = 0.420; P = 0.026) was suggested for the CPAP treated group. Of note is that when variations in pattern of alterations were controlled for BMI values, significant impact was noted in the CPAP group on the decreased association of changes between right thalamic plasticity and verbal fluency scores (r = − 0.554 vs r = − 0.345). The results of other neuroplastic adaptations and corresponding changes in cognitive domains are summarized in Supplementary Table S5.

## 4 Discussion

Our findings demonstrate that just one month of CPAP treatment, combined with psychoeducation and lifestyle modifications, can redress several cognitive and morphometric deficits in patients with moderate–severe OSA (defined with an AHI ≥ 10 events/hour). These data are consistent with the recovery of gray matter regions and cognition in patients with OSA reported by Canessa et al. (2011) to occur following three months of CPAP treatment (Canessa et al., 2011). Moreover, the same group has recently shown that appreciable recovery of cognition and white matter, including that in corpus callosum, occurs over the course of 12-month treatment with CPAP (Castronovo et al., 2014). Our study is, to the best of our knowledge, the first time that these findings have been replicated in much shorter timeframe (Gozal, 2013).

The baseline neuroanatomical and neurocognitive impairments recorded in OSA patients were comparable to previously reported neurocognitive deficits (Yaffe et al., 2014, Kumar et al., 2014, Kylstra et al., 2013, Kim et al., 2016) and involved the neurocircuitry hubs formerly shown as specifically vulnerable to chronic sleep fragmentation and associated nocturnal cycles of intermittent hypoxia in clinical and preclinical studies (Rosenzweig et al., 2013a, Rosenzweig et al., 2013b, Rosenzweig et al., 2015, Yaffe et al., 2014, Gozal, 2013). Specifically, hypotrophic changes in regions corresponding to hippocampal formation, basal ganglia and parts of corpus callosum were suggested by the neuroimaging investigations (Fig. 3). Similarly, we recorded excessive daytime somnolence and significant impairments in cognitive domains of attention, working memory, verbal episodic memory and semantic memory in our OSA patients at baseline (Table 2).

Of note is that a minimal improvement, both in cognitive and neuroanatomical measures, was demonstrated in the OSA patients randomized to BSC over one month (Table 2, Supplementary Table S3). Similar improvement in patients with OSA has been previously associated with the improved sleep quality and argued by the beneficial impact of diet, exercise, lifestyle modifications and psychoeducation (Araghi et al., 2013). In addition to any intervention effects or natural neuro homeostatic fluctuations, it is also possible that some of the cognitive changes may have represented practice effects.

In order to distinguish the beneficial impact of CPAP from that of any other interventional modality, the between-groups comparison of normalized changes was performed. Consequently, three major constructs were highlighted. Specifically, neuroplastic hypertrophic changes in thalamus, decreased daytime somnolence and improvements in verbal episodic memory were shown as more likely to result from adaptive processes driven by treatment with CPAP, and as such merit further discussion.

### 4.1 Excessive Daytime Somnolence

**Fig. 5:**
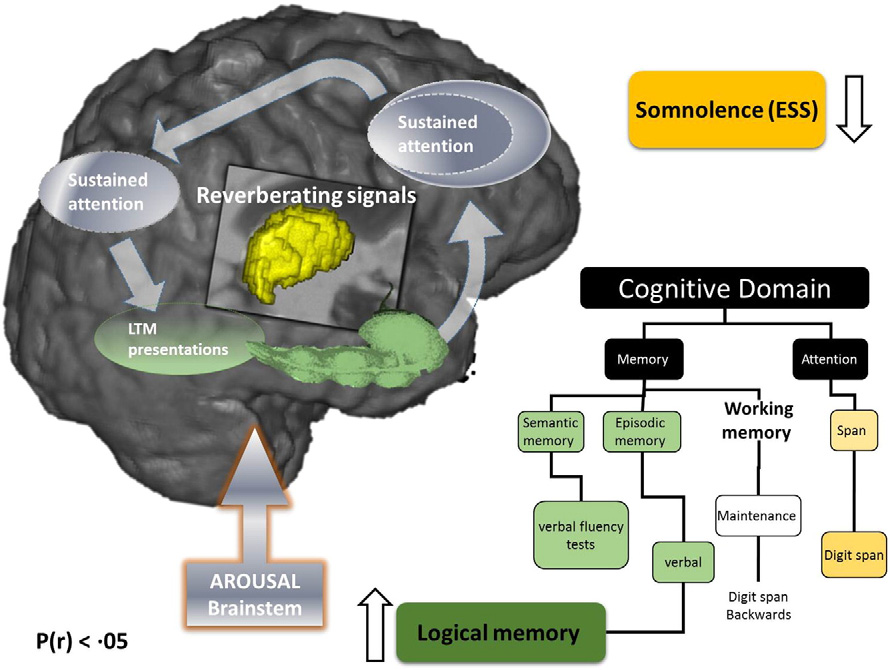
Schematic presentation of the neuroarchitecture behind working memory maintenance processes that might be implicated in improved daytime somnolence and verbal episodic memory by the CPAP treatment. Distributed nature of processes and representations involved to solve working-memory tasks is shown (McNab and Klingberg, 2008, Eriksson et al., 2015), with thalamus (central structure) acting as a functional interface between arousal and attentional regulation (Schiff, 2008). The hippocampus (elongated green), is the structure most frequently reported affected by neuroimaging in OSA (Rosenzweig et al., 2015, Rosenzweig et al., 2013b), here it is proposed to bind aspects of working and episodic memory (Eriksson et al., 2015, Axmacher et al., 2010). The list of cognitive tests used in our study to investigate impact of CPAP treatment on associated cognitive domains is also depicted. CPAP treatment leads to improvement in verbal episodic memory (green box), which may be due to the interplay of the cascade of gradual changes in the semantic and verbal working memory. In addition, CPAP leads to the improvement of excessive daytime somnolence (yellow box), which in turn appears to be significantly, correlated to ensuing brainstem alterations. Our results also suggest that comparable subjective sleepiness has significantly less impact on the attention and maintenance of working memory capacity in patients treated with CPAP. Abbreviations: CPAP: Continuous Positive Airway Pressure; BSC: best supportive care; LTM: long term memory; ESS: Epworth sleepiness scale.

Somnolence is a major complaint in OSA, and whilst its exact neurological substrate is incompletely understood (Guilleminault and Brooks, 2001), it is likely that chronic low-level neuroinflammation affects brain structures that participate in the initiation and maintenance of sleep and alertness (Rosenzweig et al., 2015). In agreement with this notion, improvement in daytime somnolence following one month of treatment with CPAP was significantly correlated with neuroplastic alterations in brainstem (Fig. 4) in our study. In addition, brainstem alterations were associated with hypertrophic thalamic changes, which were in turn closely correlated with alterations in bilateral cerebellar cortices and hippocampus (Fig. 2, Fig. 4). The neuroplastic stream of these associations correlated closely with changes in scores of tests of attentional regulation and working memory capacity (Fig. 5).

We propose that our findings represent changes in consciousness and arousal regulation that can occur within one month, perhaps primarily driven by increased activity/plasticity of brainstem pathways. Many of these pathways are known to underlie rapidly generated brief shifts of arousal at times of increased cognitive and corticothalamic demand (Schiff, 2008). It follows that our findings might also suggest specific vulnerability of a thalamocortical oscillator to sleep fragmentation and nocturnal cycles of intermittent hypoxia, something that has been long suggested by neurophysiological and neuroimaging data in OSA (Rosenzweig et al., 2013a, Rosenzweig et al., 2013b). In cases of a faulty oscillator, distributed network activity and associated memory may suffer across long-range cortico-cortical pathways, and within cortico-striatopallidal-thalamocortical loop volleys (Schiff, 2008).

It is notable that our findings also show that, whilst in untreated OSA patients changes in scores of tests of attention and working memory capacity were strongly affected by the severity of their sleepiness, this significant association was all but negated by the effects of treatment with CPAP over one month (Fig. 5). As patients with OSA have been shown to be more likely to experience a driving-related traffic accident, and given that such accidents have been shown as more likely in those who manifest greater daytime sleepiness, we argue that this finding implies strong clinical rationale for CPAP treatment even for a short duration (Malhotra et al., 2015, Rosenzweig et al., 2015, Gozal, 2013).

### 4.2 Thalamocortical Circuitry in OSA

As already suggested, recruitment of central thalamic neurons via the brainstem occurs in response to increasing cognitive demand, stress and fatigue that reduce behavioral performance (Schiff, 2008). Through thalamic activation, neurons across the cerebral cortex and striatum can be depolarized, and their activity selectively gated by descending or ascending signals related to premotor attention and alerting stimuli (Schiff, 2008).

We and others have argued that in OSA, direct injury to the thalamocortical neurons, or their prominent deafferentation as a result of multifocal, neuroinflammatory brain processes, could lead to severe impairment of prefrontal functional integration and arousal regulation (Rosenzweig et al., 2013a, Schiff, 2008, McNab and Klingberg, 2008, Portas et al., 1998). In keeping with this suggestion, we speculate that our findings show neuroplastic compensatory thalamic changes, instigated by one month of CPAP treatment, leading to a wider circuitry reorganization involving brainstem, cerebellum and hippocampal connectivity (Fig. 2, Fig. 4). We also propose that CPAP-driven adaptive processes further optimize activation of the thalamic system that acts as functional interface between arousal and attentional regulation (Portas et al., 1998, Eriksson et al., 2015). This might then underlie positive subjective experience of decreased mental effort required in order to solve tasks following the CPAP treatment. It should be noted that the results of our partial correlation analysis (Supplementary Table S5) suggest that BMI might act as a confounder in noted executive and language gains. This potential association between BMI and cognition is not a new proposition, and results from a recent prospective controlled trial of eighty patients with mild cognitive impairment suggest a strong link between BMI decrease and improvements in verbal memory, verbal fluency and executive function (Horie et al., 2016).

### 4.3 Neurocognitive Architecture of Working and Episodic Memory in OSA

One month of CPAP treatment resulted in a partial recovery of episodic and working-memory capacity. A crucial role for working memory in temporary information processing and guidance of complex behavior, as well as its impairment in OSA (Twigg et al., 2010), has been recognized (Eriksson et al., 2015). There is an emerging consensus that working-memory maintenance results from the interactions among associative memory representations and basic processes, including attention, that are instantiated as reentrant loops between frontal and posterior cortical areas, as well as subcortical structures (McNab and Klingberg, 2008, Eriksson et al., 2015). In our study, over one month, wider reorganization in a distributed memory system for associative learning occurred, e.g. thalamocortical changes were associated with changes in bilateral hippocampi and cerebellar cortices. None of these alterations were observed in the BSC group (Fig. 4, Fig. 5).

It is of further interest that the hippocampus, the structure most frequently reported affected by neuroimaging in OSA (Rosenzweig et al., 2013b, Rosenzweig et al., 2015), has recently been proposed to act as a major shared substrate for working and episodic memory (Eriksson et al., 2015). Axmacher and colleagues (2010) have suggested that the hippocampus plays a role in working-memory maintenance of multiple items by neural assemblies synchronized in the gamma frequency range, locked to consecutive phase ranges of oscillatory activity in the hippocampal theta range (Axmacher et al., 2010). Of note, a decrease in theta band has been shown to occur after apnea and hypopnea events in some patients with OSA, and CPAP has been shown to normalize EEG changes (Rosenzweig et al., 2014).

Several limitations should be considered when interpreting the findings of this study; the cross-sectional design, the absence of follow up of healthy controls and strict exclusion criteria used disallow conclusions to be made regarding causality or interactions between OSA, its treatment, and various comorbidities such as hypertension and diabetes. Also, one cannot infer which, if any, interventional aspects of the lifestyle modifications and psychoeducation, might have contributed to observed changes. Furthermore, correlations between changes in regional volumes and neuropsychological scores were exploratory and hypothesis-generating. Their interpretation of presumed complex and diverse causalities remains a challenging issue for the future. Nonetheless, we suggest that our findings indicate an important clinical message about the benefits of CPAP treatment.

The results of this study show that one month of CPAP treatment provides a sufficient timeframe for rudimentary neuroplastic changes to occur within targeted brain structures of patients with moderate to severe OSA. We also speculate that the structural changes provide a basic neurocognitive architectural scaffold for further reorganization, which underlies some of the observed functional recovery in working and episodic memory.

## Dedication

This work is dedicated to the memory of Dr Oliver Sacks, an eminent neurologist, neuroscientist and an inspiring role model and storyteller.

## Contributors

All authors contributed equally to the writing and revision of this manuscript. Data collection and analysis were carried out by IR, MG, WC, MK, MM, AM, PG, SW, MJM. The authors apologize to all the colleagues whose outstanding work could not be cited due to space limitations.

## Funding

Wellcome Trust [103952/Z/14/Z] and NIHR Respiratory and Cardiac Biomedical Research Units, at the Royal Brompton and Harefield NHS Foundation Trust and Imperial College London. The funders had no role in study design, data collection, data analysis, interpretation or writing of the report.

## Figures and Tables

**Table 1:** Baseline characteristics of participants.

	Controls	CPAP_baseline_	BSC_baseline_	P^a^ CPAP_baseline_	P^a^ BSC_baseline_	P^a^_baseline_
Age (years)	43.5 (2.1)	48.6 (1.9)	46.5 (2.2)	0.089	0.324	0.503
BMI (kg/m^2^)	27.8 (0.6)	34.7 (1.2)	34.1 (1.3)	< 0.001	< 0.001	0.707
Education (years)	15.0 (0.4)	14.8 (0.5)	14.5 (0.5)	0.720	0.495	0.755
AHI (events/h)	n/a	36.6 (5.1)	36.4 (4.0)	n/a	n/a	0.979
ODI (events/h)	2.3 (0.2)	35.4 (5.1)	37.8 (4.5)	< 0.001	< 0.001	0.655
ESS	6.2 (0.5)	13.1 (1.0)	12.4 (0.9)	< 0.001	< 0.001	0.548
Gender (male): n (%)^b^	28 (80.0%)	28 (100.0%)	24 (88.9%)	0.014	0.491	0.111
Dexterity (right-handed): n (%)	35 (100.0%)	28 (100.0%)	27 (100.0%)	n/a	n/a	n/a

Data presented as mean (SEM) or number of patients N (%). Abbreviations: Controls: healthy volunteers (N = 35); BSC: baseline values for patients treated with best supportive care (BSC) (N = 27); CPAP: baseline values for patients treated with Continuous Positive Airway Pressure (CPAP) with BSC (N = 28); BMI: body mass index, AHI: apopnea/hypopnea index. ODI: oxygen desaturation index. P columns: P CPAP_baseline_ = baseline CPAP with BSC group vs controls P BSC_baseline_ = baseline BSC vs controls; P = baseline CPAP vs BSc scores.

a Bonferroni corrected P values.

b Fisher exact test.

**Table 2:** Changes in neurocognitive function.

Cognitive tests	Controls	OSA_baseline_	BSC_baseline_	CPAP_baseline_	BSC_1_ _month_	CPAP_1_ _month_	P^a^	P^a^ BSC_1_ _month_	P^a^ CPAP_1_ _month_
Immediate LM	47.06 (1.84)	36.36 (1.32)	37.59 (1.91)	35.17 (1.84)	41.70 (2.28)	44.43 (1.99)	< 0.001	0.070	0.338
Delayed LM	29.79 (1.37)	22.25 (0.97)	23.41 (1.45)	21.14 (1.28)	27.33 (1.55)	29.93 (1.42)	< 0.001	0.238	0.946
ACE-R	94.91 (0.99)	90.55 (1.11)	88.59 (2.01)	92.42 (0.88)	90.70 (1.85)	91.86 (2.44)	0.008	0.038	0.220
Memory	24.18 (0.41)	22.05 (0.57)	21.15 (0.87)	22.92 (0.70)	23.07 (0.65)	23.79 (0.37)	0.009	0.143	0.493
Fluency	12.50 (0.3)	11.25 (0.3)	11.22 (0.43)	11.28 (0.42)	11.41 (0.49)	11.89 (0.45)	0.006	0.050	0.248
Language	24.12 (0·71)	24.18 (0.37)	23.56 (0.68)	24.78 (0.28)	23.63 (0.68)	25.11 (0.24)	0.930	0.628	0.230
SSF	9.00 (0.31)	8.33 (0.23)	8.22 (0.31)	8.42 (0.33)	7.93 (0.38)	8.18 (0.4)	0.079	0.032	0.104
SSB	8.65 (0.26)	7.65 (0.24)	7.52 (0.39)	7.79 (0.29)	7.37 (0.39)	8.18 (0.31)	0.009	0.007	0.250
DSF	12.06 (0.33)	10.22 (0.31)	10.26 (0.45)	10.18 (0.43)	10.70 (0.4)	10.32 (0.51)	< 0.001	0.011	0.005
DSB	8.18 (0.39)	6.73 (0.31)	6.93 (0.45)	6.54 (0.42)	7.41 (0.47)	7.26 (0.52)	0.005	0.210	0.157
TMA	24.12 (1.18)	27.34 (1.04)	29.19 (1.49)	25.56 (1.38)	28.02 (1.56)	24.26 (1.28)	0.049	0.047	0.937
TMB	41.23 (2.0)	62.02 (3.65)	69.76 (6.33)	54.56 (3.31)	61.53 (4.81)	51.05 (3.68)	< 0.001	< 0.001	0.017
ESS	6.18 (0.49)	12.80 (0.67)	12.44 (0.88)	13.14 (1.01)	12.11 (0.93)	8.18 (0.85)	< 0.001	< 0.001	0.038

Data presented as mean (SEM). Memory, fluency and language test scores were calculated from respective subtest scores of the Addenbrooke's Cognitive Examination-Revised (ACE-R) test. Normality was checked using the Kolmogorov–Smirnov test. The plots were normally distributed so independent sample *t*-test statistics were used to compare patient groups and controls. P columns: P = baseline OSA scores vs controls; P BSC = BSC vs controls; P CPAP = CPAP with BSC group vs controls.

Abbreviations: Controls: baseline values for healthy volunteers (N = 35). OSA: baseline values for all obstructive sleep apnea (OSA) patients before any intervention (N = 55); BSC: patients treated with best supportive care (BSC) for one month (N = 27); CPAP: patients treated with Continuous Positive Airway Pressure (CPAP) with BSC for one month (N = 28). ESS: Epworth sleepiness scale. TMB: trail making test B; TMA: trail making test A; DSF: digit-span forward task; DSB: digit-span backward task; SSF: spatial-span forward test; SSB: spatial-span backward test; ACE-R: Addenbrooke's Cognitive Examination- Revised; LM: logical memory test.

a Bonferroni corrected P values.

## Appendix A Supplementary data

Supplementary tables. Image 1
