# Supplementary material for: Changes in Neurocognitive Architecture in Patients with Obstructive Sleep Apnea Treated with Continuous Positive Airway Pressure
Source: eBioMedicine. 2016 Mar 25;7:221–9. doi: 10.1016/j.ebiom.2016.03.020 (PMC4909326; doi:10.1016/j.ebiom.2016.03.020)
Supplement: Supplementary file 1 — Supplementary tables. [file mmc1.docx]

**SUPPLEMENTARY APPENDIX**

**Supplement to: Changes in Neurocognitive Architecture in Patients with Obstructive Sleep Apnea Syndrome Treated with Continuous Positive Airway Pressure Treatment.**

Ivana Rosenzweig^1,2,3+,^MD, Martin Glasser^4+,^ MBBS, William R. Crum^1,7^,PhD, Matthew J. Kempton^1,7^, PhD, Milan Milosevic^8^, PhD, Alison McMillan^4^, PhD, Guy D. Leschziner^1,3,6^, PhD, Veena Kumari^5^, PhD, Peter Goadsby^6,7^, MD, Anita K. Simonds^4^, PhD, Steve C.R. Williams^1,7^, PhD, Mary J. Morrell^1,4^, PhD.

**TABLE OF CONTENTS**

1. **List of Tables:**

Table S1: Additional baseline characteristics of two groups of patients.

Table S2: The effect of one month of interventions on normalised volumes of structures that were registered hypotrophic at baseline.

Table S3: The effect of the Continuous Positive Airway Pressure or Best Supportive Care regimes at 1 month on changes in volumes of structures.

Table S4: The effect of the Continuous Positive Airway Pressure or Best Supportive Care regimes at 1 month on changes in cognitive domains.

Table S5: Post Hoc targeted Interregional and Cognitive Domains Differential Correlations.

1. **List of Tables**

**Table S1: Additional baseline characteristics of two groups of patient.**

| **Additional Baseline Characteristics** |  | **N** | **Mean** | **SD** | **P^a^** |
| --- | --- | --- | --- | --- | --- |
| TST | BSC | 27 | 369·39 | 71·86 | 0·042 |
|  | CPAP | 28 | 419·31 | 102·18 |  |
| Total Deep sleep | BSC | 27 | 52·32 | 31·69 | 0·684 |
|  | CPAP | 28 | 56·17 | 37·64 |  |
| total REM | BSC | 27 | 64·10 | 37·91 | 0·611 |
|  | CPAP | 28 | 69·38 | 38·58 |  |
| % deep sleep | BSC | 27 | 13·27 | 7·23 | 0·937 |
|  | CPAP | 28 | 13·11 | 7·78 |  |
| % REM | BSC | 27 | 17·49 | 9·84 | 0·537 |
|  | CPAP | 28 | 15·99 | 7·98 |  |
| Desats | BSC | 27 | 235·19 | 140·77 | 0·786 |
|  | CPAP | 28 | 248·11 | 203·96 |  |
| apnoeas/hypopnoeas | BSC | 27 | 227·78 | 134·42 | 0·802 |
|  | CPAP | 28 | 239·75 | 208·90 |  |
| AHI | BSC | 27 | 36·40 | 20·91 | 0·979 |
|  | CPAP | 28 | 36·58 | 27·15 |  |
| AI | BSC | 27 | 15·00 | 16·61 | 0·418 |
|  | CPAP | 28 | 19·73 | 25·31 |  |
| Time SaO2 <90% | BSC | 27 | 55·65 | 56·37 | 0·503 |
|  | CPAP | 28 | 69·28 | 89·10 |  |

^a^Bonferroni corrected P values. Apart from the total sleep time, the baseline characteristics were broadly similar between the two groups.

*Abbreviations*: TST: total sleep time; REM: rapid eye movement sleep; desats: deaturations; AHI: apnoea hyponea index; AI: arousal index; SD: standard deviation; C: BSC: best supportive care at baseline; CPAP: patients to be treated with continuous positive airway pressure (CPAP) at baseline.

**Table S2: The effect of one month of interventions on normalised volumes of structures that were registered hypotrophic at baseline.**

| **Structures** | **Controls** | **OSA_baseline_** | **BSC_baseline_** | **CPAP_baseline_** | **BSC_1month_** | **CPAP_1month_** | ***P^a^*** | ***P^a^BSC*_1month_** | ***P^a^CPAP*_1month_** |
| --- | --- | --- | --- | --- | --- | --- | --- | --- | --- |
| L Pallidum | 0·1094 (0·015) | 0·1026 (0·015) | 0·1027 (0·015) | 0·1025 (0·016) | 0·1045 (0·024) | 0·1067 (0·017) | 0·039 | 0·177 | 0·504 |
| L Hippocampus | 0·2355 (0·025) | 0·2244  (0·030) | 0·2192 (0·032) | 0·2295 (0·026) | 0·2290 (0·051) | 0·2381 (0·027) | 0·071 | 0·334 | 0·697 |
| R Pallidum | 0·1028 (0·015) | 0·096 (0·014) | 0·098 (0·011) | 0·0945 (0·016) | 0·0974 (0·020) | 0·0980 (0·013) | 0·031 | 0·177 | 0·172 |
| CC Mid Posterior | 0·0296 (0·006) | 0·0269 (0·005) | 0·0264 (0·004) | 0·0274 (0·006) | 0·0279 (0·006) | 0·0283 (0·006) | 0·033 | 0·270 | 0·400 |

Data presented mean (SD), number of patients N. Mean values: percentage ratios of subcortical volumes to the ICV as determined by FreeSurfer. *P* columns: *P*= OSA at baseline vs controls; *P* BSC= BSC vs controls; *P* CPAP= CPAP with BSC group vs controls. ^a^Bonferroni corrected *P* values.

*Abbreviations*: R: right; L: left; CC: corpus callosum; ICV: intracranial volume; ODI: oxygen desaturation index; SD: standard deviation; C: Controls: baseline values for healthy volunteers (N=35). OSA: baseline values for all obstructive sleep apnoea (OSA) patients before any intervention (N=55); BSC: patients treated with best supportive care (BSC) for one month (N=27); CPAP: patients treated with continuous positive airway pressure (CPAP) with BSC for one month (N=28).

**Table S3: The effect of the Continuous Positive Airway Pressure or Best Supportive Care regimes at one month on changes in volumes of structures.**

| **Structure** | **BSC N=27** | **SEM** | **CPAP N=28** | **SEM** | **P^a^** |
| --- | --- | --- | --- | --- | --- |
| L CerebellumWhiteMatter | 2·87% | (2·44%) | 4·27% | 2·93% | 0·716 |
| L CerebellumCortex | -1·13% | 1·65% | 1·37% | 2·62% | 0·428 |
| L Thalamus | -0·76% | 2·30% | 3·04% | 2·55% | 0·274 |
| L Caudate | 5·08% | 2·82% | 1·26% | 2·12% | 0·281 |
| L Putamen | 1·20% | 2·65% | 7·90% | 3·23% | 0·116 |
| L Pallidum | 3·10% | 3·10% | 7·52% | 5·49% | 0·490 |
| BrainStem | 0·49% | 2·10% | -0·11% | 2·07% | 0·840 |
| L Hippocampus | 6·73% | 3·97% | 4·84% | 2·91% | 0·701 |
| L Amygdala | -0·42% | 3·70% | 6·62% | 3·79% | 0·190 |
| R CerebellumWhiteMatter | 3·36% | 2·42% | 3·48% | 3·41% | 0·977 |
| R CerebellumCortex | -0·09% | 2·13% | 2·59% | 2·71% | 0·443 |
| RThalamus | -2·29% | 2·05% | 4·04% | 2·57% | 0·061 |
| R Caudate | 2·92% | 2·86% | 0·98% | 2·73% | 0·626 |
| R Putamen | 2·47% | 2·83% | 8·53% | 3·35% | 0·174 |
| R Pallidum | 1·10% | 3·02% | 8·69% | 6·92% | 0·325 |
| R Hippocampus | 2·60% | 2·66% | 3·51% | 3·25% | 0·831 |
| R Amygdala | 0·33% | 3·27% | 8·83% | 5·25% | 0·179 |
| CC_Posterior | 3·77% | 3·44% | -0·16% | 3·18% | 0·404 |
| CC_Mid_Posterior | 7·38% | 4·81% | 5·07% | 3·75% | 0·705 |
| CC_Central | 7·86% | 3·90% | 3·72% | 2·80% | 0·390 |
| CC_Mid_Anterior | 7·50% | 3·52% | 3·27% | 3·35% | 0·388 |
| CC_Anterior | 5·52% | 3·04% | 0·41% | 3·45% | 0·273 |

Data presented mean (SEM), controlled for ICV and normalised for differential changes (diff%) from the baseline (diff=[V/domainpre-V/domainpost]/V/domain pre x100)· ^a^Bonferroni corrected P values· Significant difference between changes in two groups of patients after one month of CPAP (with BSC) or BSC alone ·

*Abbreviations*: R: right; L: left; CC: corpus callosum; ICV: intracranial volume; SEM: standard error of mean; C: BSC: patients treated with best supportive care (BSC) for one month (N=27); CPAP: patients treated with continuous positive airway pressure (CPAP) for one month.

**Table S4: The effect of the Continuous Positive Airway Pressure or Best Supportive Care regimes at one month on changes in cognitive domains·**

| **Cognitive test** | **BSC** | | **CPAP** | | **P^a^** |
| --- | --- | --- | --- | --- | --- |
|  | **Mean** | **SD** | **Mean** | **SD** |  |
| Immediate LM | 14·28% | 39·00% | 34·78% | 48·43% | 0·090 |
| Delayed LM | 23·41% | 32·45% | 57·20% | 75·46% | 0·037* |
| ACE-R | 2·63% | 3·67% | -0·34% | 14·52% | 0·306 |
| Memory (ACE) | 11·93% | 16·49% | 5·63% | 14·79% | 0·141 |
| Fluency (ACE) | 1·17% | 10·81% | 8·14% | 25·49% | 0·195 |
| Language (ACE) | 0·45% | 6·71% | 1·45% | 4·75% | 0·523 |
| SSF | -1·87% | 21·75% | 0·46% | 30·66% | 0·748 |
| SSB | 1·03% | 26·62% | 8·46% | 27·94% | 0·317 |
| DSF | 7·40% | 23·88% | 5·50% | 33·34% | 0·810 |
| DSB | 12·43% | 37·78% | 16·22% | 53·19% | 0·762 |
| TMA | -3·11% | 18·97% | -1·35% | 24·89% | 0·771 |
| TMB | -6·99% | 21·13% | 3·17% | 53·42% | 0·361 |
| ESS | 2·46% | 39·97% | -27·97% | 46·46% | 0·012* |

Data presented mean (SD), normalised for differential changes [diff∆(%)] from the baseline (diff=[score/domainpre-score/domainpost]/score/domainpre x100). ^a^Bonferroni corrected P values. Significant difference between changes in two groups of patients after one month of CPAP (with BSC) or BSC alone.

*Abbreviations*: ESS: Epworth sleepiness scale· TMB: Trail Making Test B ;TMA: Trail Making Test A; DSF: Digit-span Forward Task; DSB: Digit-Span Backward Task; SSF: Spatial-span Forward Test; SSB: Spatial-span Backward Test; ACE-R: Addenbrookes Cognitive Examination- Revised ; LM: logical memory test;·SD: standard deviation; C: BSC: patients treated with best supportive care (BSC) for one month (N=27); CPAP: patients treated with continuous positive airway pressure (with BSC) for one month (N=28)·

**Table S5: Post Hoc targeted Interregional and Cognitive Domains Differential Correlations**

| **CPAP** N=28 | **Correlation coefficients** | | | | | |
| --- | --- | --- | --- | --- | --- | --- |
| Interregional and Cognitive Domains Differential Correlations | **R Thalamus** | | **ESS** | | **Delayed LM** | |
|  | Pearson's r | Pearson's r -partial correlation controlled to BMI | Pearson's r | Pearson's r -partial correlation controlled to BMI | Pearson's r | Pearson's r -partial correlation controlled to BMI |
| Brainstem | **0·741**** | **0·740**** | **-0·370*** | -0·368 | 0·052 | 0·058 |
| L Cerebellum Cortex | **0·770**** | **0·792**** | 0·010 | 0·008 | -0·023 | -0·043 |
| R Cerebellum Cortex | **0·750**** | **0·763**** | 0·027 | 0·018 | 0·026 | 0·016 |
| L Hippocampus | **0·490**** | **0·511**** | -0·224 | -0·243 | 0·096 | 0·081 |
| R Hippocampus | **0·510**** | **0·525**** | -0·233 | -0·253 | 0·123 | 0·108 |
| ACE-R | **-0·554**** | **-0·556**** | 0·062 | 0·064 | 0·134 | 0·137 |
| DSB | -0·230 | -0·244 | 0·295 | 0·315 | **0·451*** | **0·476*** |
| DSF | **-0·499**** | **-0·533**** | 0·224 | 0·257 | 0·230 | 0·267 |
| SSB | **-0·370*** | **-0·383*** | -0·279 | -0·270 | 0·289 | 0·308 |
| fluency ACE-R | **-0·554**** | -0·345 | 0·002 | 0·016 | **0·406*** | **0·428*** |

| **BSC** N=27 | **Correlation coefficients** | | | | | |
| --- | --- | --- | --- | --- | --- | --- |
| Interregional and Cognitive Domains Differential Correlations | **R Thalamus** | | **ESS** | | **Delayed LM** | |
|  | Pearson's r | Pearson's r -partial correlation controlled to BMI | Pearson's r | Pearson's r -partial correlation controlled to BMI | Pearson's r | Pearson's r -partial correlation controlled to BMI |
| Brainstem | 0·387 | 0·316 | 0·100 | 0·205 | 0·105 | 0·220 |
| L Cerebellum Cortex | 0·384 | 0·326 | 0·320 | 0·163 | -0·059 | 0·003 |
| R Cerebellum Cortex | 0·339 | 0·271 | 0·119 | 0·199 | -0·192 | -0·138 |
| L Hippocampus | 0·366 | 0·316 | 0·188 | 0·248 | -0·242 | -0·204 |
| R Hippocampus | **0·402*** | 0.366 | 0·107 | 0·149 | -0·120 | -0·084 |
| ACE-R | -0·022 | -0·017 | 0·260 | 0·259 | 0·370 | 0·371 |
| DSB | 0·113 | 0·107 | **0·499**** | **0·511**** | 0·280 | 0·291 |
| DSF | 0·275 | 0·275 | **0·591**** | **0·604**** | -0·081 | -0·076 |
| SSB | -0·096 | -0·115 | 0·245 | 0·258 | 0·301 | 0·316 |
| fluency ACE-R | 0·181 | 0·217 | 0·031 | 0·014 | -0·19 | -0·213 |

^a^Bonferroni corrected P values. Significant difference between changes in two groups of patients after one month of CPAP (with BSC) or BSC use. The partial correlations coefficients column (BMI controlled) describes the linear relationship between two variables while controlling for the effects of body mass index with interpretation analogous to linear regression analysis. *Denotes correlation significant at 0·05 level. ** Correlation is significant at 0·01 level.

*Abbreviations*: BMI: body mass index; ESS: Epworth sleepiness scale· TMB: Trail Making Test B ;TMA: Trail Making Test A; DSF: Digit-span Forward Task; DSB: Digit-Span Backward Task; SSF: Spatial-span Forward Test; SSB: Spatial-span Backward Test; ACE-R: Addenbrookes Cognitive Examination- Revised ; LM: logical memory test;·SD: standard deviation; C: BSC: patients treated with best supportive care (BSC) for one month (N=27); CPAP: patients treated with continuous positive airway pressure (with BSC) for one month (N=28).
